# Anti-NMDA receptor encephalitis with coexisting autoimmune GFAP astrocytopathy presenting with psychiatric symptoms leading to a suicide attempt: a case report

**DOI:** 10.1186/s12245-025-01095-9

**Published:** 2025-12-20

**Authors:** Soichiro Kano, Ryo Kamidani, Takahito Miyake, Tomohide Ando, Yuto Tamaoki, Yoya Ono, Masato Shiba, Akio Kimura, Shozo Yoshida, Takayoshi Shimohata, Hideshi Okada

**Affiliations:** 1 Department of Emergency and Disaster Medicine, Gifu University Graduate School of Medicine, Gifu, Japan; 2 Department of Pediatric Critical Care, Saitama Children’s Medical Center, Saitama, Japan; 3 Department of Neurology, Gifu University Graduate School of Medicine, Gifu, Japan; 4 Department of Emergency and Critical Care Medicine, Saitama Medical Center, Kawagoe, Japan; 5 Department of Emergency Medicine, Matsunami General Hospital, Gifu, Japan; 6 Center for One Medicine Innovative Translational Research, Gifu University Institute for Advanced Study, Gifu, Japan

**Keywords:** Glial fibrillary acidic protein astrocytopathy (GFAP-A), Autoimmune encephalitis, Psychiatric symptoms, Suicide attempt, Hyponatremia

## Abstract

**Background:**

Distinguishing altered consciousness owing to an underlying organic disorder from a psychiatric etiology can be challenging in patients presenting at the emergency department after a suicide attempt.

**Case presentation:**

The patient was a 52-year-old woman who jumped from the third floor of her house. She sustained a small subdural hematoma in the left cerebellar tentorium and a fracture of the right first rib. She was admitted to our tertiary emergency and critical care center for further evaluation of the head injury and remained clinically stable. On hospital day 2, the patient was diagnosed with severe depression by a psychiatrist and was transferred to a psychiatric ward. However, she developed disturbances of consciousness and cognitive impairment that could not be explained by psychiatric illness thereafter. Further workup revealed anti-N-methyl-D-aspartate receptor (anti-NMDAR) encephalitis and autoimmune glial fibrillary acidic protein (GFAP) astrocytopathy as the etiology. High-dose intravenous methylprednisolone therapy together with high-dose intravenous immunoglobulin was initiated. Disturbances of consciousness and cognitive impairment did not fully recover; however, she was transferred to another hospital on day 149.

**Conclusion:**

Apart from psychiatric causes, organic etiologies should be considered in patients who have attempted suicide if psychiatric interventions cannot explain cognitive abnormalities. This approach facilitates timely diagnosis and management of treatable diseases such as coexisting anti-NMDAR encephalitis and autoimmune GFAP astrocytopathy.

## Background

Patients with severe trauma often sustain high-energy injuries caused by traffic accidents, occupational accidents, or, in some cases, suicide attempts, such as jumping from heights, in the context of underlying psychiatric disorders, including depression or schizophrenia [1]. Distinguishing altered consciousness due to an underlying organic disorder from genuine psychiatric symptoms in patients presenting after a suicide attempt can be challenging, especially in emergency departments. This raises an important clinical question: whether there are treatable conditions that manifest as suicidal ideation and are not necessarily attributed to psychiatric etiology.

Autoimmune glial fibrillary acidic protein astrocytopathy (GFAP-A) is a recently established disease characterized by autoimmune meningoencephalitis or meningoencephalomyelitis [2]. Given that its first description in 2016, GFAP-A has been a treatable autoimmune encephalitis with diverse clinical manifestations, including psychiatric symptoms [3]. Here, we report a rare case of anti-N-methyl-D-aspartate receptor (anti-NMDAR) encephalitis with coexisting GFAP-A in a patient manifesting with suicidal tendencies.

## Case presentation

The patient was a 52-year-old woman with no known psychiatric history. She had undergone a radical hysterectomy for cervical cancer 22 years earlier and had contracted COVID-19 3 months before the injury. Three days before admission, she began to exhibit depressive symptoms, including loss of motivation, decreased concentration, psychomotor agitation, and suicidal ideation. On the day of admission, she jumped from the third floor of her house, sustaining multiple injuries.

At the emergency outpatient department, she was hypotensive (81/70 mmHg), tachycardic (133 bpm), afebrile (36.7 °C), and in respiratory distress (20 cpm) with 99% oxygen saturation at 10 L via non-rebreather mask. Glasgow Coma Scale score was 14 (eye, 4; verbal, 4; motor, 6). Hemodynamic instability improved after intravenous fluid resuscitation. Furthermore, laboratory tests revealed leukocytosis (32,630/µL), anemia (Hb 10.3 g/dL), elevated fibrin degradation product (84 µg/mL), and marked hyponatremia (120 mEq/L) (Table 1). Head computed tomography (CT) revealed a small subdural hematoma in the left cerebellar tentorium, and chest x-ray showed a fracture of the right first rib. A follow-up head CT on hospital day 3 showed interval reduction of the tentorial subdural hematoma, and later brain magnetic resonance imaging (MRI) revealed no expansion or chronic subdural changes. She was admitted to our tertiary emergency and critical care center for further evaluation of the head injury and remained neurologically and hemodynamically stable during the early hospitalization.

**Table 1:** Laboratory findings

Biochemistry*			Complete Blood Count*		
Total protein	5.3	g/dL	White Blood Cells	32,630	/uL
Albumin	3.2	g/dL	Red Blood Cells	3.26 × 10^6^	/uL
AST	27	IU/L	Hemoglobin	10.3	g/dL
ALT	25	IU/L	Hematocrit	29.2	%
LDH	285	IU/L	Platelet	2.3 × 10^5^	/uL
ALP	33	IU/L			
Total bilirubin	1.0	mg/dL	Coagulation Status*		
Creatinine	1.34	mg/dL	APTT	22	s
BUN	15.4	mg/dL	PT-INR	1.08	
Sodium	120	mEq/L	Fibrinogen	178	mg/dL
Potassium	4.7	mEq/L	FDP	84	µg/mL
Chloride	88	mEq/L			
Calcium	8.5	mg/dL	Cerebrospinal Fluid**		
Glucose	320	mg/dL	Total protein	99	mg/dL
CRP	0.02	mg/dL	Glucose	63	mg/dL
			Adenosine deaminase	9	IU/L
			Total cell count	17	/3 µL
			Mononuclear cells	17	/3 µL
			Polymorphonuclear cells	0	/3 µL

On hospital day 2, she was diagnosed with severe depression by a psychiatrist and was transferred to a psychiatric ward. Over the course of admission, she developed apraxia associated with upper limb tremors and delusions on day 8. Furthermore, hyponatremia persisted and was managed as central salt-wasting syndrome (CSWS) on day 9 with fludrocortisone and sodium chloride supplementation. Despite treatment, cognitive impairment, psychiatric symptoms, and hyponatremia did not improve. Respiratory status and consciousness rapidly deteriorated, requiring intensive care unit admission, tracheal intubation, and mechanical ventilation on day 30.

**Fig. 1:**
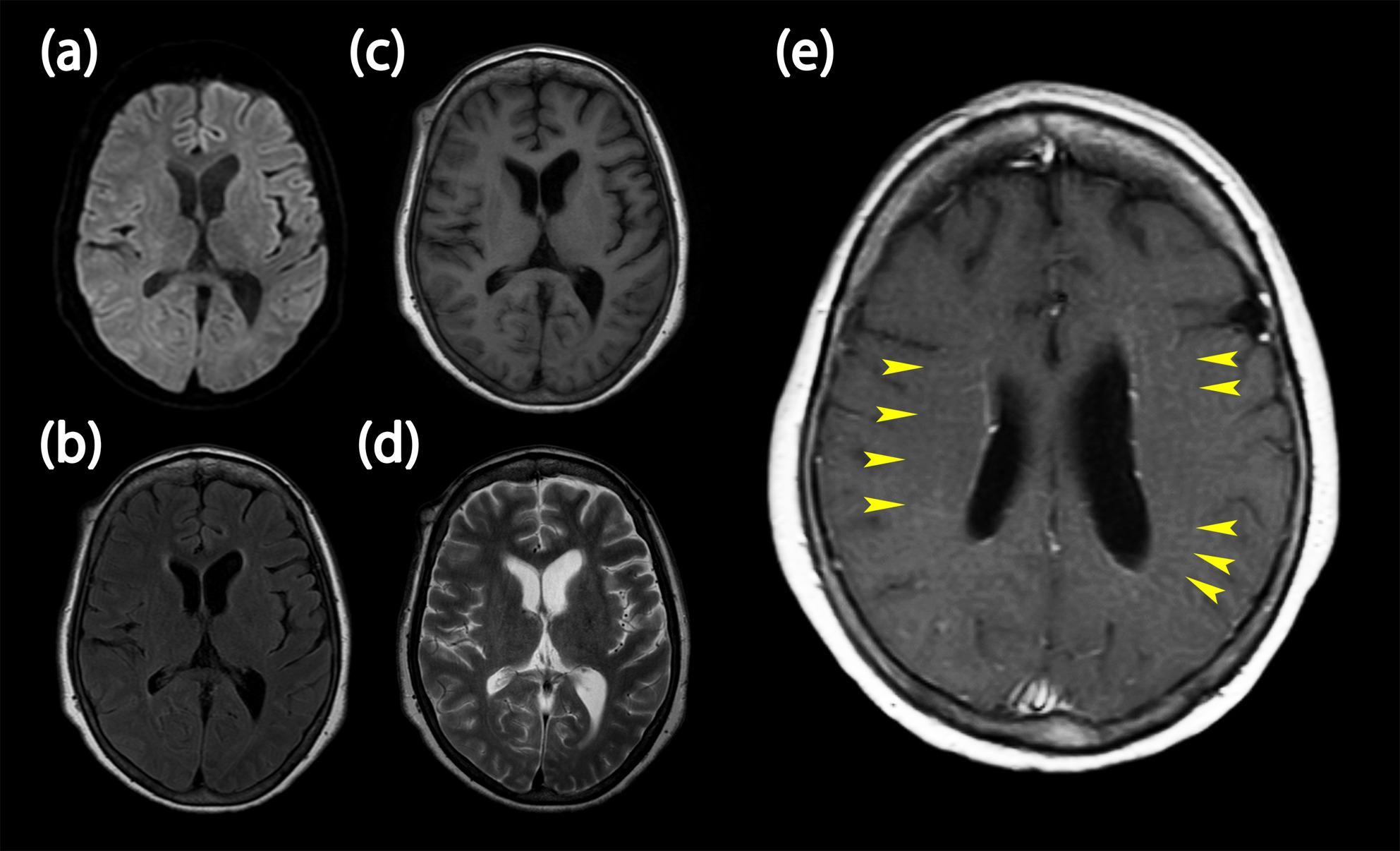
Brain MRI showing linear perivascular enhancement around the lateral ventricles. Brain MRI obtained on day 30: (**a**) diffusion-weighted imaging (DWI), (**b**) fluid-attenuated inversion recovery (FLAIR), (**c**) T1-weighted, (**d**) T2-weighted, and (**e**) contrast-enhanced T1-weighted sequences. Yellow arrowheads indicate the characteristic linear perivascular (radial) enhancement extending from the lateral ventricles, which is typical of GFAP astrocytopathy

**Fig. 2:**
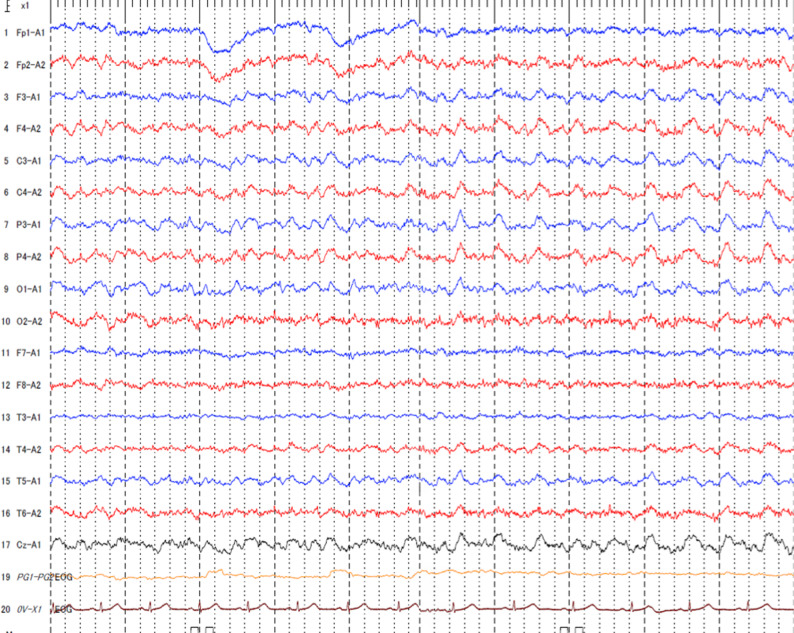
Electroencephalography (EEG) demonstrating generalized rhythmic delta activity. EEG shows generalized rhythmic delta activity (2–4 Hz) with posterior predominance and intermittent 8–9 Hz alpha activity, without epileptiform discharges

Cerebrospinal fluid (CSF) examination on day 24 revealed elevated total protein (99 mg/dL), normal glucose (63 mg/dL), and mild pleocytosis (17 cells/3 µL, all mononuclear). CSF adenosine deaminase level was within the normal range (9 IU/L). In addition to routine CSF testing, oligoclonal band and CSF LGI1 antibody tests were performed, but were negative. Additionally, tumor screening, including chest X-ray, contrast-enhanced CT of the chest and abdomen, whole-body 18 F-FDG PET-CT, and pelvic MRI, was performed to search for underlying tumors such as ovarian teratoma; however, no neoplastic lesions were identified. On day 30, anti-GFAPα antibodies were detected in the CSF using cell-based assay (CBA) and tissue-based assay, and contrast-enhanced MRI demonstrated linear enhancement extending from the lateral ventricles on T1-weighted images (Fig. 1a–e). On day 35, anti-NMDAR antibodies were also detected using CBA. An EEG on hospital day 35 demonstrated generalized rhythmic delta activity (2–4 Hz) with posterior predominance and intermittent 8–9 Hz alpha activity, without epileptiform discharges, consistent with diffuse encephalopathy (Fig. 2).

Accordingly, she was diagnosed with anti-NMDAR encephalitis with coexisting GFAP-A, and high-dose intravenous methylprednisolone (IVMP) together with high-dose intravenous immunoglobulin (IVIg) was initiated. Hyponatremia and respiratory failure improved following immunotherapy; although consciousness did not improve, she remained unresponsive to verbal stimuli. The persistent impairment of consciousness was attributed to anti-NMDAR encephalitis, and multiple courses of IVMP and rituximab were administered. Clinical improvement was limited, and hence, she was transferred to another hospital on day 149. Her mental status stabilized thereafter, and she was able to eat independently, although episodes of disorientation, impaired attention, and psychomotor slowing persisted.

## Discussion

This case is noteworthy given that a patient who attempted suicide was ultimately diagnosed with an underlying treatable organic disorder. In the emergency setting, it is easy to assume that psychiatric manifestations equate to primary psychiatric illness. However, when abnormalities such as impaired consciousness or cognitive decline cannot be fully explained by psychiatric symptoms alone, clinicians must consider organic causes. This approach aligns with prior recommendations that emphasize recognizing autoimmune encephalitis in acute psychiatric presentations [4]. In our case, the rapid progression from depressive symptoms to apraxia, tremor, and disorientation—together with persistent hyponatremia and later respiratory failure—could not be explained by primary psychiatric disease, prompting further neurological evaluation. The traumatic tentorial subdural hematoma was unlikely to have contributed to the later neurological deterioration, as follow-up CT on hospital day 3 demonstrated interval reduction and subsequent MRI showed neither expansion nor chronic subdural formation. Therefore, the subacute decline was considered more consistent with autoimmune encephalitis rather than trauma-related progression. An autoimmune encephalitis, including the recently recognized GFAP-A entity, represents a critical differential diagnosis in this context.

GFAP-A was first described in 2016 and typically presents with fever, headache, and generalized fatigue, followed within weeks by altered mental status, gait disturbance, tremor, and psychiatric symptoms [2]. It remains a relatively rare disorder [5]. Apraxia, as seen in our patient, has been described in GFAP astrocytopathy and in anti-NMDAR encephalitis; however, it appears to be an infrequent manifestation compared with other cognitive and behavioral changes [6]. The diagnosis is supported by the presence of anti-GFAP antibodies in CSF and the characteristic linear perivascular enhancement around the ventricles on MRI, both of which were seen in the present case [7]. The patient was also positive for an anti-NMDAR antibody, raising the possibility of an overlap syndrome.

Previous reports have indicated that GFAP-A may coexist with anti-NMDAR encephalitis, and when such overlap occurs, the clinical phenotype often resembles that of anti-NMDAR encephalitis [8]. In published overlap cases, the clinical picture is typically dominated by anti-NMDAR encephalitis with prominent psychiatric symptoms, seizures, or movement disorders. In contrast, GFAP-A contributes radial perivascular enhancement and, in some patients, hyponatremia or meningoencephalomyelitis [9, 10]. Our patient fits this anti-NMDAR–predominant pattern, but is unusual in that the initial manifestation was a suicide attempt with traumatic injury. Furthermore, in patients presenting with acute or subacute psychiatric symptoms without a personal or familial psychiatric history, exclusion of anti-NMDAR encephalitis is essential [11]. In this regard, the psychiatric manifestations in our patient may have been driven predominantly by anti-NMDAR encephalitis. The present case presented with psychiatric symptoms that led to a suicide attempt, a presentation that, to our knowledge, has been rarely documented in the literature.

Another important aspect of this case is hyponatremia. In autoimmune encephalitis, whether hyponatremia represents syndrome of inappropriate antidiuretic hormone secretion (SIADH) or CSWS remains controversial. Autoimmune encephalitis, such as GFAP-A, frequently present with hyponatremia [5, 12]. Therefore, inpatients with psychiatric manifestations accompanied by unexplained hyponatremia, autoimmune or inflammatory encephalitis should be considered as a differential diagnosis. Considering that this patient also developed persistent hyponatremia, it is plausible that the coexistence of GFAP-A contributed to the severity and atypical nature of her depressive state. From a practical perspective in emergency medicine, clinicians should promptly recognize and correct hyponatremia while investigating potential organic causes.

Plasma exchange (PE) may be considered in autoimmune encephalitis; however, the evidence supporting its use in GFAP-A is limited [13]. Similarly, although PE is sometimes used in anti-NMDAR encephalitis, there is no consensus on its role as standard therapy [14]. In this case, partial improvement in hyponatremia and autonomic instability had already begun following IVMP and IVIg, and further intensification of immunotherapy was deemed unlikely to provide substantial additional benefit. Therefore, rituximab was prioritized instead of PE.

This case represents an overlap of anti-NMDAR encephalitis and GFAP-A. From an emergency medicine standpoint, the critical message is not which entity predominated, but rather the importance of recognizing that treatable autoimmune encephalitis can underlie psychiatric symptoms, even when a suicide attempt is the presenting event. Early immunological evaluation and timely initiation of therapy are crucial to improving patient outcomes.

This case report has some limitations. First, a full paraneoplastic syndrome-related antibody panel was not performed; therefore, a paraneoplastic process cannot be entirely excluded. Second, although chest/abdominal CT, whole-body PET-CT, and pelvic MRI showed no evidence of malignancy, additional age-appropriate screening, such as colonoscopy, mammography, and breast ultrasonography, was not conducted. The absence of these investigations should be considered when interpreting the diagnostic workup.

In conclusion, this case demonstrates that anti-NMDAR encephalitis with coexisting GFAP-A, a recently established and treatable autoimmune encephalitis, can initially present with psychiatric symptoms leading to suicide attempts. Physicians should consider autoimmune encephalitis in patients with unexplained psychiatric manifestations or altered consciousness, as timely recognition allows for effective immunotherapy.

## Data Availability

All data generated or analyzed during this study are included in this published article. Further inquiries can be directed to the corresponding author.

## Abbreviations

CSF: cerebrospinal fluid
GFAP: glial fibrillary acidic protein
IVMP: intravenous methylprednisolone
IVIg: intravenous immunoglobulin
MRI: magnetic resonance imaging
NMDAR: N-methyl-D-aspartate receptor
